# Triboelectric Nanogenerator for Droplet Energy Harvesting Based on Hydrophobic Composites

**DOI:** 10.3390/ma16155439

**Published:** 2023-08-03

**Authors:** Yang Zheng, Jingjing Li, Tiantian Xu, Hongzhi Cui, Xiaoyi Li

**Affiliations:** 1 College of Materials Science and Engineering, Ocean University of China, Qingdao 266100, China; zhengyang@stu.ouc.edu.cn (Y.Z.); ljj7568@stu.ouc.edu.cn (J.L.); xtt2027@163.com (T.X.); cuihongzhi@ouc.edu.cn (H.C.); 2 Key Laboratory for Special Functional Materials of Ministry of Education, School of Materials Science and Engineering, Henan University, Kaifeng 475004, China

**Keywords:** hydrophobic composite film, triboelectric nanogenerator, hydrophobic electrode, droplet energy

## Abstract

Triboelectric nanogenerators (TENG) have shown great potential in harvesting energy from water. For the TENG that harvests water energy, surface hydrophobicity is crucial for its performance. In this paper, we prepare a hydrophobic composite film of Polyvinylidene Fluoride/Polydimethylsiloxane/Polytetrafluoroethylene (PVDF/PDMS/PTFE) and an electrode of Polyaniline/Carbon nanotubes/Silver nanowires (PANI/CNTs/AgNWs) by electrospinning technology and a doping method, respectively, which are served as the friction layer and top electrode of TENG. The contact angle of the hydrophobic film and electrode both reach over 120°, which makes the separation process between water and the interface complete and promotes the output of TENG. The open-circuit voltage (V_oc_) and short-circuit current (I_sc_) can reach 150 V and 60 μA approximately. In addition, the composite electrode can be applied in the preparation of complex electrode shapes. Furthermore, the different reactions of TENG to different liquids indicate that it may contribute to liquid-type sensing systems. This work presents an efficient approach to fabricating hydrophobic films and electrodes, laying a foundation for the development of TENG for harvesting water energy.

## 1. Introduction

With the continuous development of the social economy, people’s demand for electric energy is increasing. As the main source of electricity generation, fossil fuels are non-renewable and diminishing. Therefore, there is an urgent need for sustainable energy alternatives. Water resources are abundant on Earth, encompassing a wide range of sources such as ocean energy and raindrop energy. These resources hold immense potential for harnessing renewable energy and providing sustainable solutions to meet our growing energy needs. Currently, one of the most common methods for harvesting water resources is through the construction of hydroelectric power stations. However, this approach does have certain limitations, such as geographical location, a high cost, and difficulty in collecting low-frequency water energy. The triboelectric nanogenerator (TENG) reported in 2012 shows great development potential in collecting water resources [1,2,3]. The TENG has demonstrated significant advantages in terms of its ability to harvest low-frequency energy, its ability to operate across unrestricted geographical locations and its low cost. These advantages make TENGs a promising complementary technology for electromagnetic power generation [4,5,6,7,8]. Furthermore, a TENG has a good performance in many aspects such as self-drive sensing, blue energy, and high-voltage power supply [9,10,11,12]. In terms of self-drive sensing, a TENG is capable of autonomously sensing and responding to its surroundings, allowing it to adapt to different environmental conditions. This feature enables a TENG to effectively harness energy from sources such as human motion, wind, and vibrations, ensuring optimal energy conversion and harvesting [13,14,15,16,17].

The research on water-driven TENGs focuses on two aspects: structural design and the study of surface charge transfer mechanisms [18]. Water-driven can be classified into multiple structures. The solid–liquid-based TENG has garnered significant attention due to its ability to directly interact with water, leading to a substantial reduction in friction loss. This unique characteristic has attracted considerable interest for harnessing energy from water-based sources more effectively [19,20,21,22]. Several structures of a solid–liquid-based TENG have been studied successively, including a single-electrode mode, double-electrode mode, etc. [23,24,25]. Xu et al. reported that a droplet-based electricity generator with a novel structure has attracted a lot attention due to its high-output performance, which has become the mainstream structure of a solid–liquid-based TENG [26,27]. Since the surfaces of solid–liquid-based TENG devices are in direct contact with water, the hydrophobicity of friction layers is highly required. Lots of research has reported that the hydrophobicity of the friction layer and electrodes plays a vital part in the performance of TENGs, which is contributed to by the thorough contact-separation process enhancing its charge transfer [28,29,30]. Wang et al. prepared a superhydrophobic FEP film based on a droplet electricity generator, and it was found that a TENG with film performs better [31]. When droplets and the TENG surface separate in time after contact, a layer of liquid film is formed on the surface, which will affect the output of the TENG. This fully demonstrates the influence of the hydrophobicity of the friction layer on the output performance of TENG [32]. At present, most methods for preparing hydrophobic materials use laser etching, template etching, electrochemical deposition, and chemical etching in chemistry [33,34]. However, most of these methods have the disadvantages of high cost and a complex preparation process, which is not good for large-scale use. Here, we prepared a hydrophobic film and investigated the effects of different components on its surface morphology, hydrophobicity, and triboelectricity. Furthermore, a hydrophobic electrode was prepared and we explored the influence of different conductive substances on the hydrophobicity and output performance applied to the TENG. The I_sc_ and V_oc_ of a TENG based on the hydrophobic film and an electrode can reach 60 μA and 150 V, respectively. This work may provide a guide for the preparation of hydrophobic films and hydrophobic electrodes, and increase the value of TENG in collecting water energy, such as raindrop energy and ocean energy.

## 2. Materials and Methods

### 2.1. Fabrication of PVDF/PDMS/PTFE Hydrophobic Film

A weight of 4 g PVDF solid particles, 8 g acetone solution, and 8 g N, N-dimethylformamide solution (DMF) were placed in a 30 mL sample bottle successively, and heated in a water bath at 60 °C and 200 rad/min for 2 h until the PVDF solution was completely dissolved. The PVDF solution with a certain viscosity can be used as the electrospinning solution for electrospinning. At the same time, 3 g PDMS precursor, 0.3 g PDMS curing agent, and 13.2 g tetrahydrofuran (THF) were stirred at room temperature for 30 min to make the solution evenly mixed, without agglomeration, and transparent. Then, PVDF and PDMS solution was put into the sample bottle in a ratio of 3:1 and stirred at room temperature for 10 min at the rotational speed of 200 rad/min to obtain the PVDF/PDMS precursor solution. After that, PTFE powder was added to the PVDF/PDMS solution, stirring for 12 h at the temperature of 60 °C and the rotating speed of 200 rad/min to obtain PVDF/PDMS/PTFE electrospinning precursor solution.

### 2.2. Fabrication of Hydrophobic PANI/CNTs/AgNWs Electrode

A weight of 1 g PANI was dissolved in 5 g N, N-dimethylformamide (DMF) under the conditions of 80 °C and 200 rad/min. CNTs dispersion and AgNWs were separately placed in two centrifuge tubes for dispersing 5 min ultrasonic treatment at 25 °C and 40 kHz (CNTs, C139872, Aladdin, Delaware, IA, USA). After that, dispersed CNTs and PANI were mixed at magnetic stirring for two hours. Then, dispersed AgNWs were added to evenly mixed PANI/CNTs at a volume ratio of 1:1 to obtain PANI/CNTs/AgNWs solution. The mixed liquid of PANI/CNTs/AgNWs was coated on the glass substrate (other substrates can also be applied). After drying at room temperature, the liquid was heated for 10 min at 200 °C, and then the conductive and hydrophobic material of PANI/CNTs/AgNWs was obtained after cooling at room temperature.

### 2.3. Measurements and Characterizations

Droplet release height was varied from 10 cm. The volume of water droplets were 50 μL, approximately. The I_sc_ and V_oc_ of TENG were measured by Keithley 6514 electrometer (Keithley Instruments, Cleveland, OH, USA). Raman data were recorded at a 325 nm excitation wavelength using a laser confocal microscopy Raman spectrometer. The laser wavelength range of FTIR spectroscopy was from 400 to 4000 cm^−1^.

## 3. Results and Discussion

**Figure 1:**
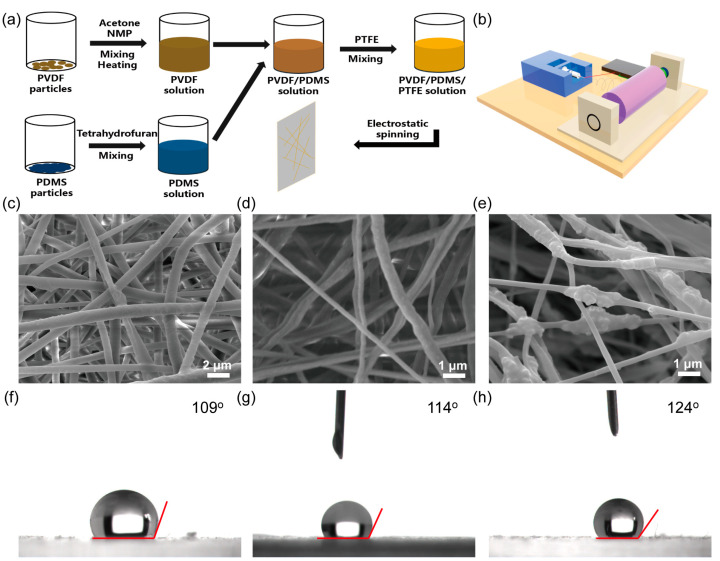
(**a**) Preparation process of the composite hydrophobic film. (**b**) Schematic diagram of electrostatic spinning device. SEM image and contact angle of (**c**,**f**) PVDF, (**d**,**g**) PVDF/PDMS, and (**e**,**h**) PVDF/PDMS/PTFE.

The preparation process of the PVDF/PDMS/PTFE precursor fluid and electrostatic spinning device is shown in Figure 1a,b. The specific preparation process is detailed in the experimental procedures section. Scanning electron microscope images of PVDF electrospun films showed that the fibers were evenly distributed, with a good morphology, and no granular material was observed, as exhibited in Figure 1c. To further enhance the hydrophobicity of PVDF films, PDMS was selected as the hydrophobic enhancing material. As can be seen from Figure 1d, the coarseness of PVDF/PDMS fibers is relatively uniform, and there is no adhesion phenomenon. The surface of a single fiber shows uneven fluctuations and a small size, and it was speculated that PDMS were distributed in PVDF fibers after THF volatilization and curing. PTFE particles were added into PVDF/PDMS solution to further enhance the triboelectric performance of the film. As shown in Figure 1e, there are tiny particles on the surface of the fibers, and we assume that these tiny particles are PTFE, which could play an important role in increasing the hydrophobicity and electron-trapping capability. The insets of Figure 1c,d show the contact angles of electrospun films with different components. Among them, Figure 1c inset shows the hydrophobicity of pure PVDF electrospun films. Because PVDF itself has certain hydrophobicity, the film prepared by electrospinning also maintains its original hydrophobic property. Figure 1d inset shows the contact angle of PVDF/PDMS electrospun film, which has the highest contact angle and the best hydrophobic performance. Combined with the SEM of PVDF/PDMS film, it is known that PDMS is evenly dispersed in the electrospinning solution by magnetic stirring before forming PVDF/PDMS films through electrospinning, which are dispersed in the interior and surface of the fibers in the form of many tiny aggregate structures in the process of electrospinning. After vacuum drying, stable micro/nanostructures can be formed and dispersed in the film, thus enhancing the roughness of the film and enhancing the hydrophobicity. The addition of PTFE has no evident influence on hydrophobicity because PTFE particles enlarged the size of the aggregate structure, which counteracts the hydrophilic property of PTFE. The addition of PTFE aims to enhance the triboelectric property owing to its strong electron-generating capacity.

**Figure 2:**
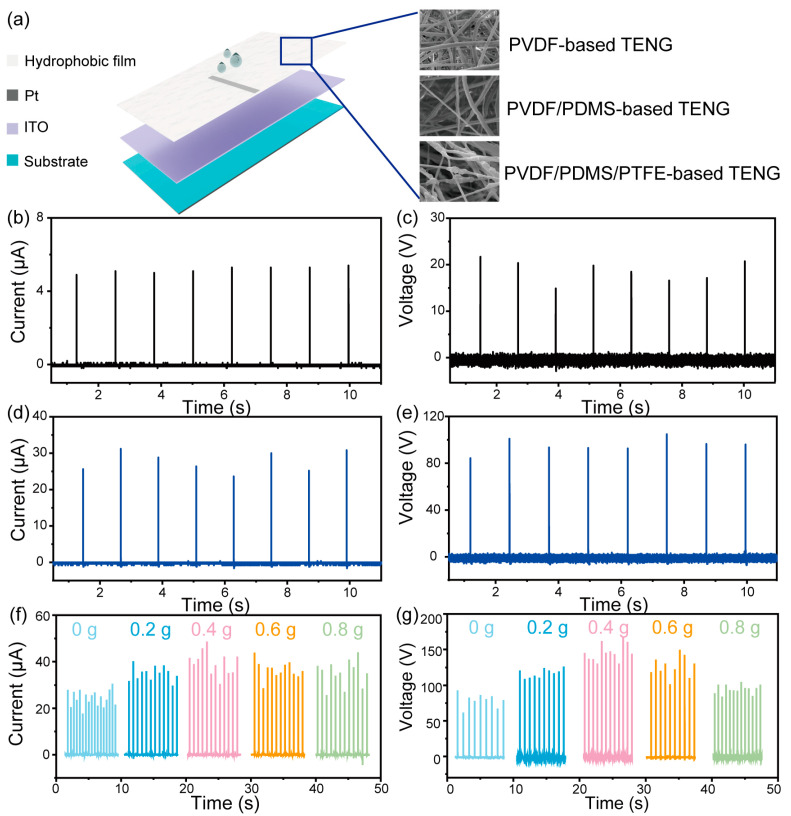
(**a**) Schematic diagram of TENG. The I_sc_ and V_oc_ of (**b**,**c**) the PVDF-TENG-based TENG, (**d**,**e**) the PVDF/PDMS-based TENG, and (**f**,**g**) the PVDF/PDMS/PTFE-based TENG.

Therefore, three TENG devices based on three different films were prepared and the performances of the TENGs were compared, as shown in Figure 2. The structure of the TENG is illustrated in Figure 2a. Pt wire and indium tin oxide (ITO) were adopted as the top and the bottom electrode, respectively. Three electrospinning films were prepared for acting as the friction layer of the TENG. The tilt angle of the TENG was set at 30° and the water droplets were released from a height of 20 cm. In order to verify the triboelectric performance of the fiber skeleton PVDF, the I_sc_ and V_oc_ test was conducted on the TENG prepared from pure PVDF thin films, as shown in Figure 2a,b. The V_oc_ and I_sc_ values are approximately 20 V and 5 μA, respectively. This is because the hydrophobic performance of pure PVDF is limited, which affects the output performance of TENG.

**Figure A1:**
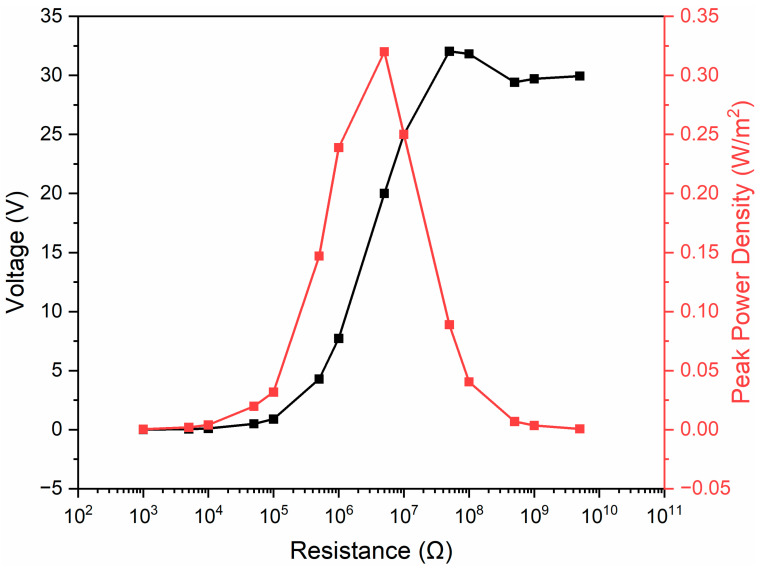
The peak power density of a TENG.

To further verify the influence of hydrophobicity on the solid–liquid-based TENG, we prepared a TENG device based on PVDF/PDMS electrospinning film and tested its output. We can see from Figure 2c,d that the V_oc_ and I_sc_ values can reach 60 V and 13 μA, respectively. Compared with pure PVDF, the output performance is remarkably improved, which further proves that increasing the hydrophobicity can improve the output performance of the TENG. Furthermore, to increase the output performance of the TENG, PTFE particles possessing a strong electron-accepting ability were added. Figure 2f,g exhibits the influence of various PTFE contents on the TENG performance. The results show that the output performance of the PVDF/PDMS/PTFE-based TENG was relatively improved compared with that of the TENG without PTFE particles, due to the high electronegativity of PTFE. However, the output of the TENG decreased slightly with the increase in PTFE content, which resulted from the reduction in the contact angle. The power density was shown in Figure A1. On the one hand, the increase in PTFE can increase the tribological properties of the film. At the same time, the slight decrease in hydrophobicity can increase the contact area of water droplets and film without affecting its contact separation at the same time. Therefore, the charge induced by water droplets and the film will increase. On the other hand, after reaching the maximum value, due to the decrease in hydrophobicity, the separation between water droplets and the friction layer is limited, and the output performance of the TENG will decrease even if the triboelectric performance increases gradually. According to previous research, for an instantaneous structure drop-based TENG, the key to generating a high current signal is the thorough contact-separation process of the waterdrop and top electrode, which places great demands on the hydrophobicity of the friction film. Furthermore, the electronegativity difference between water and film is another important factor influencing the output performance. In some cases, the two factors mentioned above may work against each other, and it is necessary to explore a balance between them.

**Figure 3:**
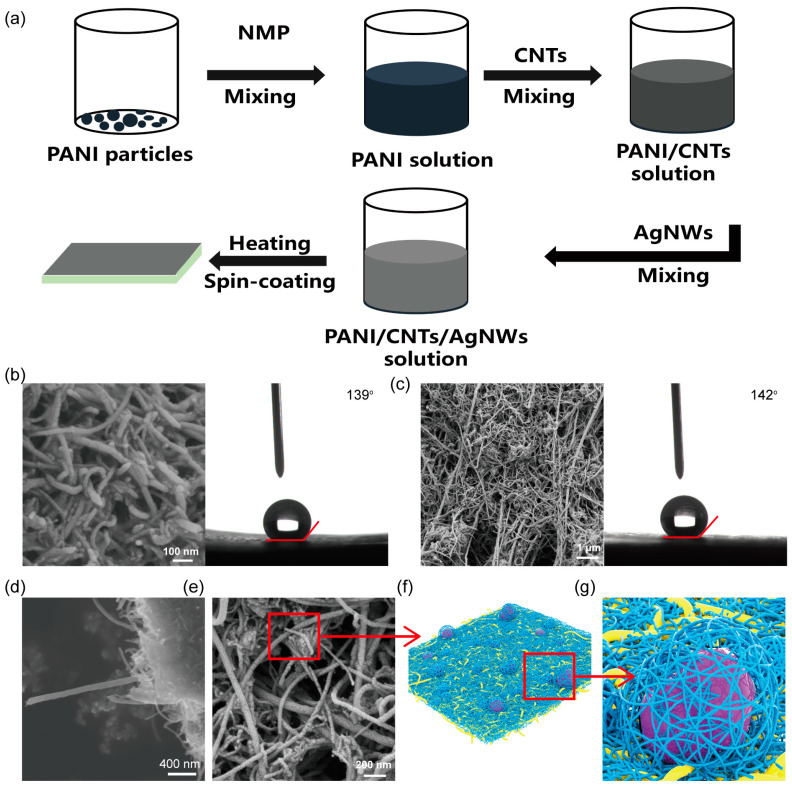
(**a**) Preparation process of the composite hydrophobic electrode. (**b**) SEM images and contact angle of PANI/CNTs. (**c**,**e**) SEM images and contact angle of PANI/CNTs/AgNWs composite electrode. (**d**) SEM images of CNTs. (**f**,**g**) Model diagram of the composite hydrophobic electrode.

In the solid–liquid-based nanogenerator, the hydrophobic electrode is also an important component in addition to the hydrophobic surface. Water droplets transfer the accumulated charge instantaneously to the bottom electrode when they contact the top electrode. If the hydrophobicity of the top electrode is relatively low, water droplets will remain on the top electrode, affecting the charge transfer. In addition, solid-state electrodes, such as silver wire, are mostly adopted in the preparation of TENG. However, these electrodes are expensive and cannot be used in complex circuits. We prepared a hydrophobic composite electrode composed of AgNWs, CB, and PANI, and the preparation process is exhibited in Figure 3a, with more details in the Experimental Procedures Section. Figure 3c,e presents the morphology of the PANI/CNTs/AgNWs composite material at different magnifications. The finer fibers were CNTs, which were wound around the surface of PANI and dispersed throughout the material. As shown in Figure 3d, the diameter of a single tube is approximately 100 nm. The presence of PANI makes CNTs interweave on their surface, which makes CNTs contact more closely, thus enhancing the conductive property of the material. In addition, PANI is tightly wrapped by CNTs, which can form micro- and nano-scale processes. Moreover, this structure is more stable and not easily damaged, thus enabling the material to achieve hydrophobic properties. The coarser fiber in the SEM image is AgNWs, and it has very strong conductivity. Dispersed in the whole system, AgNWs can enhance the conductivity of the whole material without affecting the hydrophobicity of the material. To more intuitively express the relationship between the three in the SEM image, three-dimensional graphics were established, as shown in Figure 3f,g. In the figure, the yellow part is the AgNWs, which constitute the entire conductive network. The blue part is the CNTs, which are distributed in the conductive network formed by AgNWs. The purple part is the PANI particles, which are wrapped and attached by CNTs on the surface and dispersed in the entire composite material. The enlarged image in Figure 3g shows the relationship between the three more clearly and directly.

**Figure 4:**
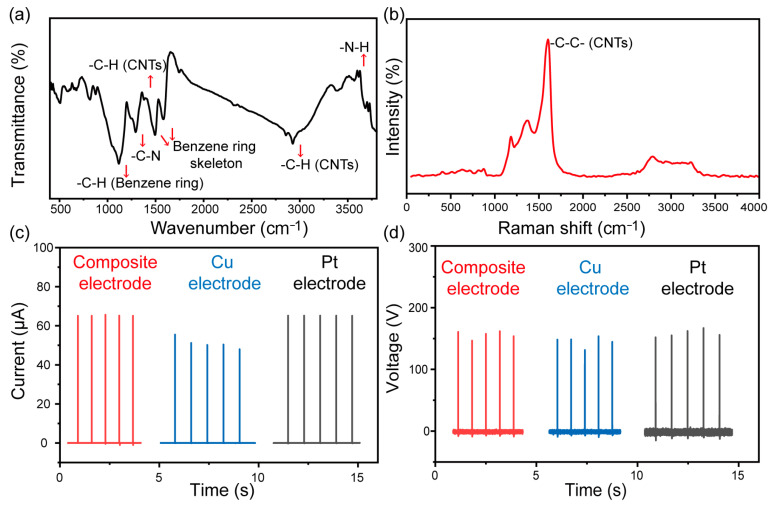
(**a**) The structures of PANI/CNT/AgNW were given by FTIR spectrum and selected polymers were scanned from 4000 to 400 cm^−1^. (**b**) Raman spectra of PANI/CNT/AgNW. (**c**) I_sc_ and (**d**) V_oc_ of PANI/CNT/AgNW-TENG.

Furthermore, to verify the electrodes contained in PANI/CNTs/AgNWs composites, Fourier transform infrared (FTIR) analysis and Raman spectroscopy were employed to investigate the three composites. As presented in Figure 4a, according to analysis, the absorption peak of 1117 cm^−1^ is for the C-H bending vibration of the benzene ring. The absorption peak of 1494 cm^−1^ and 1580 cm^−1^ are for the benzene ring skeleton. The absorption peak of 1291 cm^−1^ is for the PANI’s C-N stretching. The wave peaks of 3568 cm^−1^ and 3614 cm^−1^ are for the -N-H stretching vibration of the PANI, and the absorption peak of 1379 cm^−1^ is the -C-H bending vibration of the CNTs. The absorption peak of 2927 cm^−1^ and 2856 cm^−1^ are the -C-H stretching of the CNTs. According to Raman spectrum analysis (Figure 4b), it was proved that the existence of the -C-C- stretching vibration of the major functional groups of PANI and CNTs were not destroyed. Through the combination of the two spectra, it is sufficient to prove that there was no chemical reaction between the PANI and CNTs, and no new chemical bonds and new chemical substances were formed. Therefore, the hydrophobic and conductive properties formed by PANI/CNTs/AgNWS composites result from the properties of the material itself. Furthermore, we utilized the composite electrode to prepare the TENG acting as the top electrode. For comparison, two TENG devices with a Cu wire and Pt wire as the top electrodes were prepared, respectively. As shown in Figure 4c,d, the results illustrate that the V_oc_ and I_sc_ of the PANI/CNT/AgNW-based TENG are approximately equal to that of the Pt-based TENG and slightly higher than that of the copper electrode. We can elucidate from the results that the PANI/CNT/AgNW hydrophobic electrode can not only replace expensive platinum electrodes but also shows great advantage in complex electrode preparation, promoting the development of a diversified structure and low-cost TENG.

**Figure 5:**
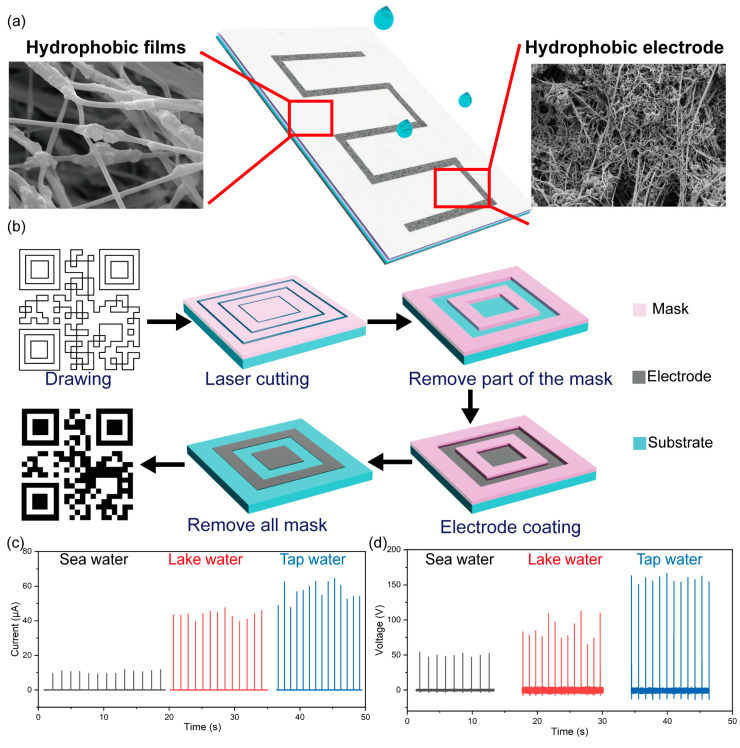
(**a**) Application of hydrophobic film and electrode to TENG. (**b**) Flowchart of the preparation of the complex-shape electrode. The I_sc_ (**c**) and V_oc_ (**d**) of different liquid types.

**Figure A2:**
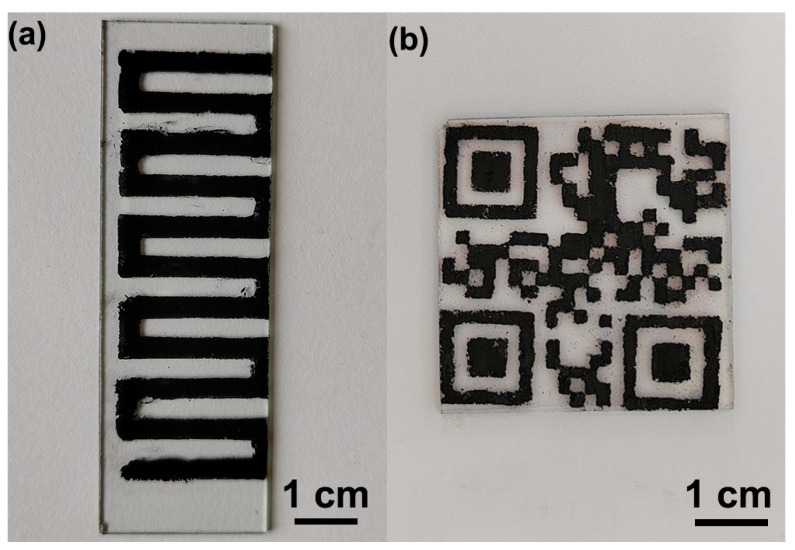
(**a**) Interdigital electrode and (**b**) code prepared by a hydrophobic electrode.

**Figure A3:**
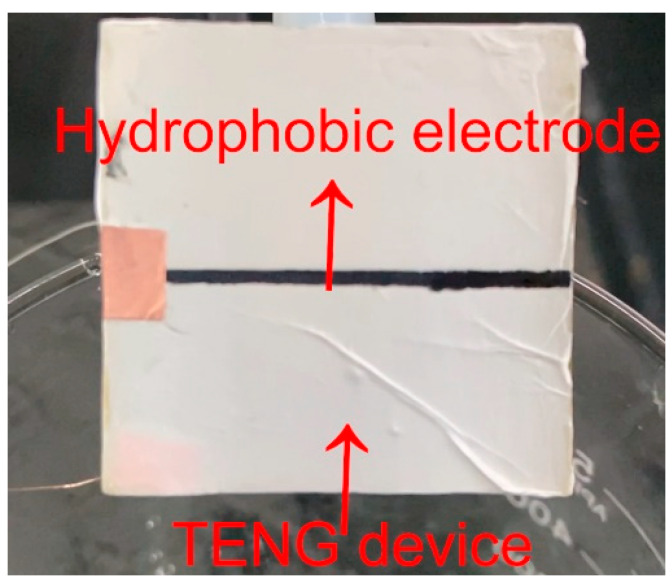
Photograph of the TENG device (Video S1: Video of water droplets driving TENG device operation).

The PANI/CNT/AgNW composite electrode has a fluidity to a certain degree. Therefore, as a hydrophobic electrode, it can prepare complex shapes for large-scale preparation. Due to this property, we can prepare a TENG device with electrodes with complex shapes that fit the friction layer. To verify that it can be made into any shape, a two-dimensional code and grid pattern were fabricated; see details in Figure A2. As Figure 5b shows, the process of preparation can be summarized as follows. First, using CAD drawing software (2020.01.00.01), a two-dimensional code and grid patterns can be drawn, and be imported into the laser cutting machine for pattern cutting. A mask is then pasted on the required basement. After cutting, the two-dimensional code and grid pattern mask is removed to obtain the two-dimensional code and grid pattern. The PANI/CNT/AgNW composite material is coated on a two-dimensional code and grid pattern by an adjustable coater. The hydrophobic electrode with complicated shapes is obtained at heating at 200 °C for 10 min after drying at room temperature and removing the remaining mask. Another approach to do this is to cut out the required mask primarily and then cover it on the required substrate for electrode coating and fabrication. The thickness of the electrode can be adjusted according to the thickness of the mask, or any pattern can be drawn through CAD to prepare the hydrophobic electrode suitable for the application. The waterdrop-based TENG can be utilized to detect liquid types, using the electrical outputs for various kinds of water, including tap water, lake water, and seawater. As shown in Figure A3, droplet TENG device was fabricated by hydrophobic electrode and hydrophobic membrane. As shown in Figure 5c,d, tap water produces a high-output current and voltage, and seawater produces the smallest output. For the sea and lake water, high ion concentrations and other impurities lead to the formation of a shielding layer, decreasing the charge induction and transfer. The reaction of TENG to different liquids indicates that TENG has application potential in the field of self-driven sensing systems.

## 4. Conclusions

In conclusion, we successfully prepared a hydrophobic composite film of PVDF/PDMS/PTFE using electrospinning technology, and a hydrophobic composite electrode of PANI/CNT/AgNW using a doping method, respectively. Furthermore, the composite film and electrode were applied in a solid–liquid-based TENG device as the friction layer and the top electrode, and the influence of a material’s composition and hydrophobicity on its output performance were studied. For the TENG harvesting water energy, the hydrophobicity of the surface and electrode is the key to promoting the output performance of the TENG. In addition, complex-shape electrode shapes can be fabricated using the composite electrode. Finally, TENG can be applied in a liquid sensing system according to the different reactions of various liquid types. Our work demonstrates a new concept in fabricating hydrophobic materials and guides the application of TENGs in sensing systems.

## Data Availability

Data available on request due to restrictions e.g., privacy or ethical.

## Supplementary Materials

The following supporting information can be downloaded at: https://www.mdpi.com/article/10.3390/ma16155439/s1, Video S1: Video of water droplets driving TENG device operation.
